# Expressive intent, ambiguity, and aesthetic experiences of music and poetry

**DOI:** 10.1371/journal.pone.0179145

**Published:** 2017-07-26

**Authors:** Elizabeth Hellmuth Margulis, William H. Levine, Rhimmon Simchy-Gross, Carolyn Kroger

**Affiliations:** 1 Department of Music, University of Arkansas, Fayetteville, Arkansas, United State of America; 2 Department of Psychological Science, University of Arkansas, Fayetteville, Arkansas, United States of America; 3 Department of Psychology, Michigan State University, East Lansing, Michigan, United States of America; University of Zurich, SWITZERLAND

## Abstract

A growing number of studies are investigating the way that aesthetic experiences are generated across different media. Empathy with a perceived human artist has been suggested as a common mechanism [1]. In this study, people heard 30 s excerpts of ambiguous music and poetry preceded by neutral, positively valenced, or negatively valenced information about the composer's or author’s intent. The information influenced their perception of the excerpts—excerpts paired with positive intent information were perceived as happier and excerpts paired with negative intent information were perceived as sadder (although across intent conditions, musical excerpts were perceived as happier than poetry excerpts). Moreover, the information modulated the aesthetic experience of the excerpts in different ways for the different excerpt types: positive intent information increased enjoyment and the degree to which people found the musical excerpts to be moving, but negative intent information increased these qualities for poetry. Additionally, positive intent information was judged to better match musical excerpts and negative intent information to better match poetic excerpts. These results suggest that empathy with a perceived human artist is indeed an important shared factor across experiences of music and poetry, but that other mechanisms distinguish the generation of aesthetic appreciation between these two media.

## Introduction

Ambiguity, or the capacity to sustain multiple interpretations, has been identified as a central characteristic of art [2]–[5]. Studies in the visual domain have produced contradictory findings, some suggesting that ambiguity elevates artistic appreciation [6], others suggesting that a moderate level of ambiguity is preferred [7], and still others suggesting that artistic appreciation increases when ambiguity is reduced or eliminated [8], [9]. But these studies have used elements like referential titles and stylistic statements to disambiguate, targeting the cognitive underpinnings of aesthetic appreciation.

Aesthetic appreciation also depends on expressive interpretation—suppositions about the artist’s emotional and communicative intent [10], [11]. For many artistic domains, such as music and poetry, significant ambiguity can characterize this expressive dimension. In music, certain structural features (fast tempi and the major mode) are known to correlate with perceptions of happiness, and others (slow tempi and the minor mode) are known to correlate with perceptions of sadness [12]–[14]. Hunter, Schellenberg and Schimmack [15] demonstrated that by mixing these cues (pairing fast tempi with the minor mode, or slow tempi with the major mode), excerpts can elicit mixed emotional responses—higher happiness ratings and higher sadness ratings for the same piece. In poetry, such expressive ambiguity is commonplace [5], [16]. How might expressive ambiguity of this sort affect aesthetic experience?

Studies of cognitive ambiguity have often used verbal information (such as a title or an explanatory statement) to alter the perceived ambiguity that is present. Yet the degree to which such information can be integrated into the experience of a work of art, and the mechanisms that underlie this integration, remains unknown. Verbal information about whether a performer was a professional or a student has been shown to affect preference for musical performances [17], with participants tending to prefer the performance they were told was by a professional. Verbal information about the content of the music has been shown to affect enjoyment of excerpts [18], with the mere presence of verbal information reducing reported enjoyment, but a similar study using children as participants showed little effect of content descriptions on enjoyment [19]. Verbal information about the content of the music—in the form of English subtitles presented during an opera screening—reduced the amount of perceived expressivity, as measured by continuous ratings provided as the music progressed, but had no effect on perceived expressivity as measured by post-excerpt responses [20].

Although potential overlaps between music and speech processing have been a central topic in music perception research [21] only some of this work has looked at the way music and linguistic information might be integrated, or the way that music, fiction, and poetry might share cognitive bases. The issue of domain specificity in aesthetic processing has been raised by Jacobsen [22]–[24] and specialized models of aesthetic appreciation have been generated for visual art [25], [26], as well as for music [27], [28]. Following an approach articulated by Jacobsen et al. [29], a recent paper by Knoop et al. [30] examines the adjectives most commonly used to describe aesthetic experiences of literature, both in general and for specific genres, including poetry, and compares them with the adjectives most commonly used to describe aesthetic experiences in other domains, including visual art and music, using data from Jacobsen et al. [29], Istok et al. [31] and Augustin et al. [32]—finding substantial overlap between music and poetry, among other findings, leading to speculation about shared features between the two, including the lack of a narrative plot as well as both domains' dependence on sound to convey emotion.

In an overview addressing the question of whether shared mechanisms might underpin affective impact in music and literature, Omigie [1] points to imagined empathy and predictive processes as the likeliest candidates. Neuroimaging studies demonstrating the activation of emotional-empathy related brain structures during reading [33], [34] have resulted in the fiction feeling hypothesis, which postulates that more emotionality in narratives results in stronger feelings of empathy [35]. A similar theory exists for music, following work by Steinbeis and Koelsch [36] that showed that brain areas involved in empathy were more strongly activated by music that participants were told had been composed by a human artist rather than a computer. Egermann and McAdams [37] found that preference for musical excerpts correlates with the degree of empathy they elicit. Moreover, participants evaluating a piece of visual art performed differently if asked to experience it from their own perspective compared to one that imposed a theory of mind task on participants: imagining the perspective of a fictitious artist whose life and attitudes had previous been summarized [38].

The psychology and philosophy of aesthetics have often distinguished between perceived and felt emotions—that is, the recognition that a particular work is (for example) expressive of sadness versus the actual induction of this emotion in the listener, viewer, or reader [39]. Drawing attention to the expressive intent of the person who created the artwork might make it likelier for a perceiver to adopt an empathetic stance, resulting in emotions that are felt in addition to merely perceived [40].

The degree to which this experience of felt rather than merely perceived emotions is pleasant might depend on whether the emotions are sad or happy. People have puzzled for centuries over the question of why people like to listen to sad music or read sad poetry [41]. Research on the enjoyment of sad music is summarized by Sachs, Damasio, and Habibi [42]. Building on data from Taruffi and Koelsch [43], Schubert [44] developed a theory of the enjoyment of sadness in aesthetic contexts. A recent paper by Brattico et al. [28] examined the neural underpinnings of this phenomenon, and Menninghaus et al. [45] investigated the enjoyment of sad literature. Aesthetic framing can affect the perceived valence of experiences of disgust [46] and anger [47],and the experience of being moved can affect the valence of experiences of sadness during film viewing [48], [49] and music listening [50]. Most laboratory studies show that people prefer happy to sad music [51], [52], although this preference disappears in certain circumstances, such as when the music is presented incidentally to a difficult task [53]. Given the potential overlap between affective mechanisms in music and literature, the question arises of whether happy artworks might also be preferred in literary domains such as poetry.

The research reported here tackles three areas of interest at once: comparative aesthetics; the relationship between extrinsic information and aesthetic experience; and responses to aesthetic ambiguity. Participants listened to 30 s excerpts of music or poetry previously categorized as expressively ambiguous—that is, excerpts that could be understood as positively or negatively valenced. They were told that information existed about the composer or poet’s intentions for each excerpt. This information was presented on screen before each excerpt. One-third of the excerpts were prefaced by intent information that was negative valenced. One-third were prefaced by intent information that was positively valenced. One-third were prefaced by information that was neutral in valence. Which participant heard which excerpt paired with which description was systematically varied using a Latin Squares design. The same descriptions used to preface the musical excerpts for one half of participants were used to preface the poetry excerpts for the other half, and vice versa. After each excerpt, participants reported how happy each excerpt was, how sad it was, how much they enjoyed it, how moving they found it, and how well the excerpt conveyed the composer or author’s intention. The construct of being moved has been well-investigated by Kühnast et al. [54] and Menninghaus et al. [55].

From the perspective of comparative aesthetics, this study seeks to understand whether extrinsic information about the artist’s intent affects aesthetic appreciation similarly for musical and poetic excerpts. Whereas poetry uses words with semantic meaning as material, music's semantic resonances are famously vague [3]. If expressive disambiguation affects aesthetic appreciation similarly for the two artistic media, it would suggest that the relationship between aesthetic appreciation and perceived expressive valence operates in a domain-general way, not dependent on the nature of the semantics employed by the medium. If it affects aesthetic appreciation differently in poetry and music, it would suggest that the material of the medium influences this relationship.

From the perspective of investigating the relationship between extrinsic information and aesthetic experiences, this study asks whether information about an artist’s expressive intent can influence the way a piece of music or poetry is processed affectively. If positively valenced information leads participants to experience excerpts as happier, and negatively valenced information leads participants to experience excerpts as sadder, it would suggest that people can integrate verbal information provided before an aesthetic experience into their emotional processing of the art. If the presentation of positively or negatively valenced information impacts the evaluation of the excerpt’s enjoyability or movingness, then it would suggest that aesthetic experiences can be at least in part a function of extrinsic information about an excerpt’s emotional tenor, demonstrating a role for cultural messaging beyond the intrinsic content of a work of art.

From the perspective of the relationship between ambiguity and aesthetic appreciation, this study investigates whether people prefer the rich multiplicity of meanings in an expressively ambiguous excerpt, or the direct communication of an excerpt that is unambiguously positive or negative. By varying the description paired with individual excerpts, this study manipulates the ambiguity of music and poetry while controlling for the actual content of the excerpts. If people prefer and are more moved by excerpts when they are prefaced by neutral intent information, it would suggest that people value the presence of expressive ambiguity in aesthetic experiences. If people prefer and are more moved by the excerpts when they are prefaced by a positive or negative description, it would suggest that people value aesthetic experiences that arise out of excerpts with a single expressive cast.

## Materials and methods

### Participants

The participants in this study were 118 students (37 male) recruited from general psychology classes at the University of Arkansas. Their mean age was 19.5 (*SD* = 3.1). Four were music majors, and one was an English major. They reported listening to an average of 16.4 hours of music each week (*SD* = 15.7), and reading poetry for an average of 2.1 hours each week (*SD* = 5.3). They volunteered to participate in exchange for partial fulfillment of a course research requirement.

### Materials & apparatus

Expressively ambiguous excerpts of music and poetry were selected as the primary stimuli of interest for this study. A smaller number of expressively unambiguous excerpts (clearly positively or negatively valenced) were selected to enhance believability of the description-excerpt pairings.

The music excerpts, listed in S1 Appendix, were drawn from the stimuli used by Hunter, Schellenberg, and Schimmack [15]. Their stimuli were excerpts of approximately 30 s that either straightforwardly conveyed positive or negative affect via consistent structural cues (major mode and fast tempo for positive; minor mode and slow tempo for negative) or conveyed ambiguous affect by mixing structural cues (major mode and slow tempo or minor mode and fast tempo). These stimuli spanned a variety of musical styles, but all were instrumental excerpts featuring no lyrics or vocal part. Of Hunter et al.'s excerpts, we used four positive, four negative, and 18 ambiguous excerpts, selected as likely to be unfamiliar to a population of college students.

Like the music excerpts, the poetry excerpts, listed in S2 Appendix, were selected from both classics (e.g., Walt Whitman) and contemporary sources (e.g., *The New Yorker*), and were edited to last approximately 30s. These excerpts were read and recorded by a professional actor instructed to speak with a neutral, affectively uninflected tone. Forty candidate recorded poetry excerpts were presented to a group of *n* = 28 participants who did not participate in the main study. They were asked to rate positivity, negativity, ambiguity (between positivity and negativity), familiarity, and enjoyment for each; of the excerpts people reported to be most unfamiliar, we selected the four most-positive, four most-negative, and 18 most-ambiguous to use in the main study.

Descriptions, listed in S3 Appendix, were written for the music and poetry excerpts to convey positive intentions (e.g., *The author/composer wrote this poem to express his passion and devotion for his love*), negative intentions (e.g., *The author/composer wrote this piece to express mourning over the death of a family member*), and neutral intentions (e.g., *The author/composer wrote this poem to experiment with different writing techniques*).

In the principal manipulation of interest, ambiguous excerpts were paired with positive, negative, or neutral intent descriptions. To preserve believability, positive excerpts were paired only with positive or neutral intent descriptions, and negative excerpts were paired only with negative or neutral descriptions. These excerpts were included to enhance believability of the description-excerpt pairings, not as comparisons of interest. Six different lists of description-excerpt pairings for the ambiguous excerpts were created using a Latin square design. For each group of participants, half the descriptions were paired with poetry excerpts, and half with music excerpts. For the next group of participants, the pairings were reversed, that is, the descriptions that had been paired with poetry excerpts for the previous group were paired with music excerpts, and vice versa. Across the entire experiment, then, the same descriptions were used for both poem and music excerpts, but for no single participant was the same description used twice.

### Design

Each participant experienced positive, negative, and neutral music and poetic excerpts, along with positive, negative, and neutral intention descriptions. Each participant was randomly assigned to one of the six lists of stimulus pairings. For the critical ambiguous excerpts, the design was a 2 (excerpt type: music, poetry) × 3 (intention description: positive, negative, neutral) repeated-measures study.

### Procedure

Participants were tested individually in a 4' × 4' booth (WhisperRoom Sound Isolation Enclosure; MDL 4848E/ENV). MediaLab software [56] was used to present instructions and intention descriptions visually on a 22" Dell P2212H monitor, and to present excerpts auditorily over Sennsheisser HD 600 open-air, around-ear headphones, as well as to collect responses via a computer keyboard. This study was approved by the University of Arkansas Institutional Review Board (protocol #15-04-664). Before each session, participants provided written informed consent by signing a form.

Once they proceeded to the experimental session, participants first answered a set of demographic questions. Then they were presented with a block of poetry excerpts or a block of music excerpts. The order of presentation of these two blocks was randomized for each participant.

At the start of each block participants were told that they would hear a number of poetry (or music) excerpts. Then they were told that these poems (or pieces of music) “are special, because in each case we know a fact about the author’s (or composer’s) intent or circumstances while writing them. For each excerpt, we’ll tell you this fact before presenting the poem (or piece).” They were informed that they should try to pay as close attention as possible, and that questions would follow each excerpt. Next, they performed a full practice trial.

Each of the 52 experimental trials started with the onscreen presentation of the intent description. Next, the recording of the poetry or music excerpt was played while the description remains onscreen. Finally, five questions were presented in the same order, each requiring the participant to select a response along a 7-point scale (1 = not at all; 7 = maximally):

- How happy did this excerpt seem?
- How moving did this excerpt seem?
- How sad did this excerpt seem?
- Did the excerpt match the intent or circumstances of the composer?
- How much did you enjoy this excerpt?

Within each experimental block (poetry or music), the individual trials were presented in random order.

## Results

### Data exclusion

Due to an error in preparing the experiment, data for one of the positive music stimulus excerpts were not recorded.

### Modeling & analytic details

Linear mixed modeling of dependent measures was carried out with the R [57] package *lme4* [58] using maximum likelihood estimation. Both participants and stimuli were treated as random-effects variables. We first fit models with maximal random-effects structure that included random slopes for each of the fixed factors within each participant and stimulus [59]. If the maximal model failed to converge, the random-effects structure was simplified incrementally by removing one random slope at a time, the one that explained the least variance in the model that did not converge. Where *p*-values are reported, they are based on *df* estimated using Satterthwaite's approximation implemented by the *lmerTest* package [60], and reported rounded to the nearest tenth.

### Excerpt selection & description checks

Verifying that the participants were paying attention and judging affect similarly to participants in previous experiments and in the norming study, happiness ratings were much higher for positive excerpts (M = 5.37, SE = 0.13) than for negative excerpts (M = 1.79, SE = 0.11), t(17.9) = 15.64, p < .001, and sadness ratings were much higher for negative excerpts (M = 5.04, SE = 0.12) than for positive excerpts (M = 1.73, SE = 0.10), t(18.2) = 16.5, p < .001; these effects did not interact significantly with excerpt type (i.e., poetry vs music). Music (M = 3.87, SE = 0.12) was rated as significantly more happy than poetry (M = 3.11, SE = 0.11), t(15.6) = 3.24, p = .005, and music (M = 3.21, SE = 0.11) was numerically though not significantly less sad than poetry (M = 3.75, SE = 0.11), t(17.6) = 1.60, p = .13.

#### Intent description match for positive & negative excerpts

Verifying that the positive and negative excerpts respectively matched positive and negative intention descriptions better than ambiguous excepts matched any kind of intention description, match ratings between positive excepts and intentions (M = 5.21, SE = 0.14) and between negative excerpts and intentions (M = 5.46, SE = 0.15) were much higher than for ambiguous excerpts and any kind of intention description (M = 3.95, SE = 0.09); for positive vs ambiguous, t(93.6) = 8.67, p < .001, and for negative vs ambiguous, t(74.7) = 10.05, p < .001. These match effects did significantly interact with excerpt type (i.e., poetry vs music), F(2, 83.7) = 3.37, p = .04, reflecting that negative poetry excerpts paired with negative intentions were rated as especially well-matched relative to ambiguous excerpts. There was no significant difference between music and poetry on match ratings overall, confirming that the intent descriptions were equally well-suited to the music and poetry samples.

#### Intent description match for ambiguous excerpts

Finally, there was significant variability in how well the intention descriptions were perceived to match the critical ambiguous excerpts. The difference in match across intention descriptions was significant, F(2, 176.5) = 3.40, p = .04, with slightly higher match ratings for positive intention descriptions (M = 4.05, SE = 0.10) than for negative intention descriptions (M = 3.94, SE = 0.10) and for neutral intention descriptions (M = 3.89, SE = 0.10). Match scores for music (M = 4.10, SE = 0.12) were slightly higher than for poetry (M = 3.82, SE = 0.12), F(1, 44.0) = 4.03, p = .051, although this difference was not significant. The interaction between intention description type and excerpt type (i.e., poetry vs music) was very strong, F(2, 125.0) = 132.39, p < .001, reflecting that for music, positive and neutral intention descriptions are better matched than negative descriptions, but the opposite pattern emerges for poetry. Because of this interaction, after presenting the effects of the critical factors of intention description and excerpt type on the aesthetic outcome measures of enjoyment, happiness, sadness, and movingness, we consider the possibility that perceived match between intention description and stimulus mediates aesthetic experience.

### Aesthetic experience

For the analyses presented in this section, only ratings provided for ambiguous excerpts are analyzed. They were examined as a function of intention description and excerpt type, as well as their interaction.

#### Enjoyment

**Fig 1:**
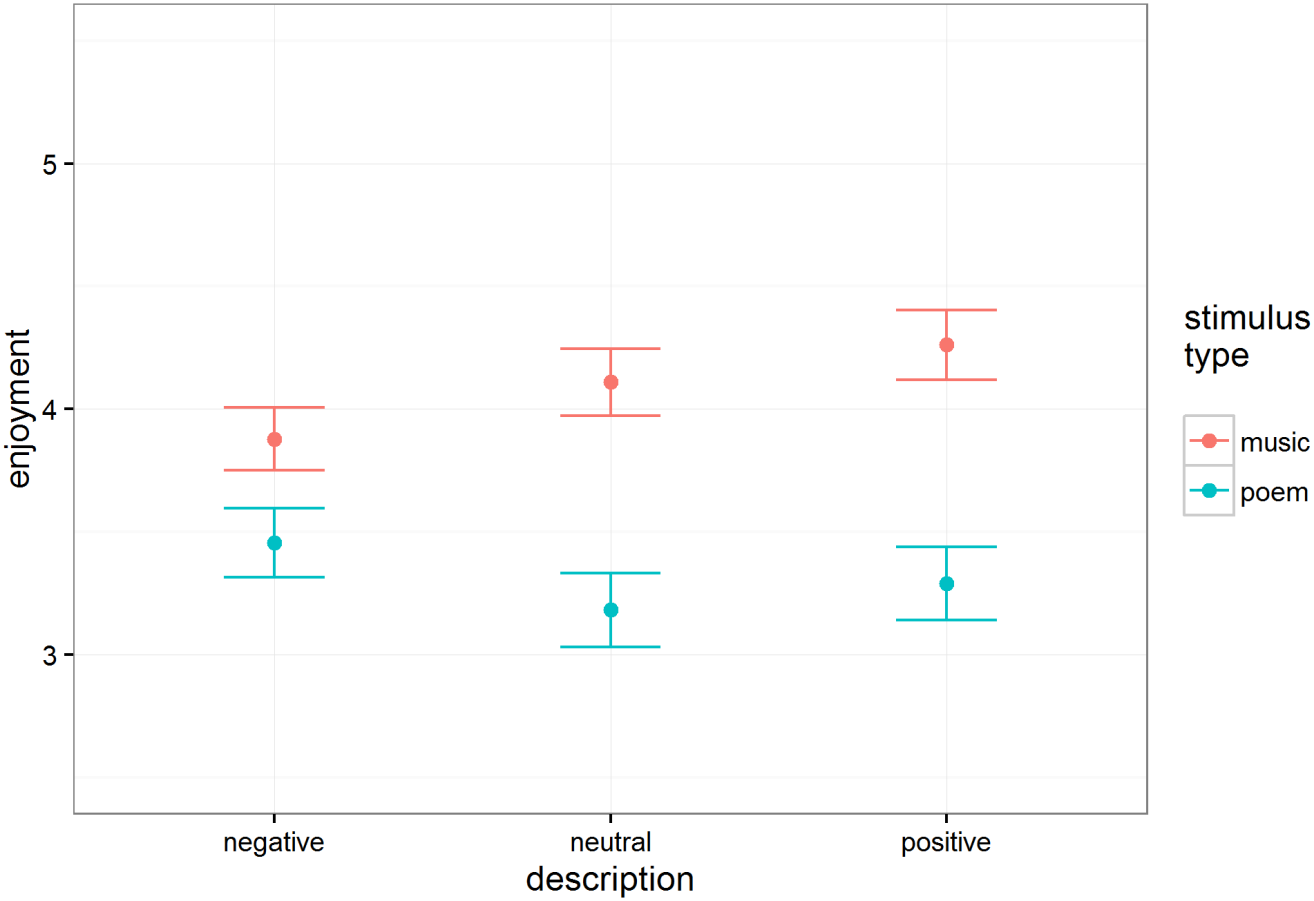
Estimated mean enjoyment ratings (n = 118 participants) as a function of intention description and stimulus type. Error bars are standard errors.

Enjoyment ratings appear in Fig 1. There was no significant effect of intention description type on enjoyment, F(2, 66.9) = 1.93, p = .15, but there was a clear interaction of intention and excerpt type, F(2, 54.9) = 15.51, p < .001; negative intentions increased enjoyment of poetry relative to the neutral descriptions, but decreased enjoyment of music relative to the neutral condition. There was also a large enjoyment advantage for music (M = 4.08, SE = 0.13) over poetry (M = 3.31, SE = 0.14), t(49.8) = 5.08, p < .001.

#### Happiness

**Fig 2:**
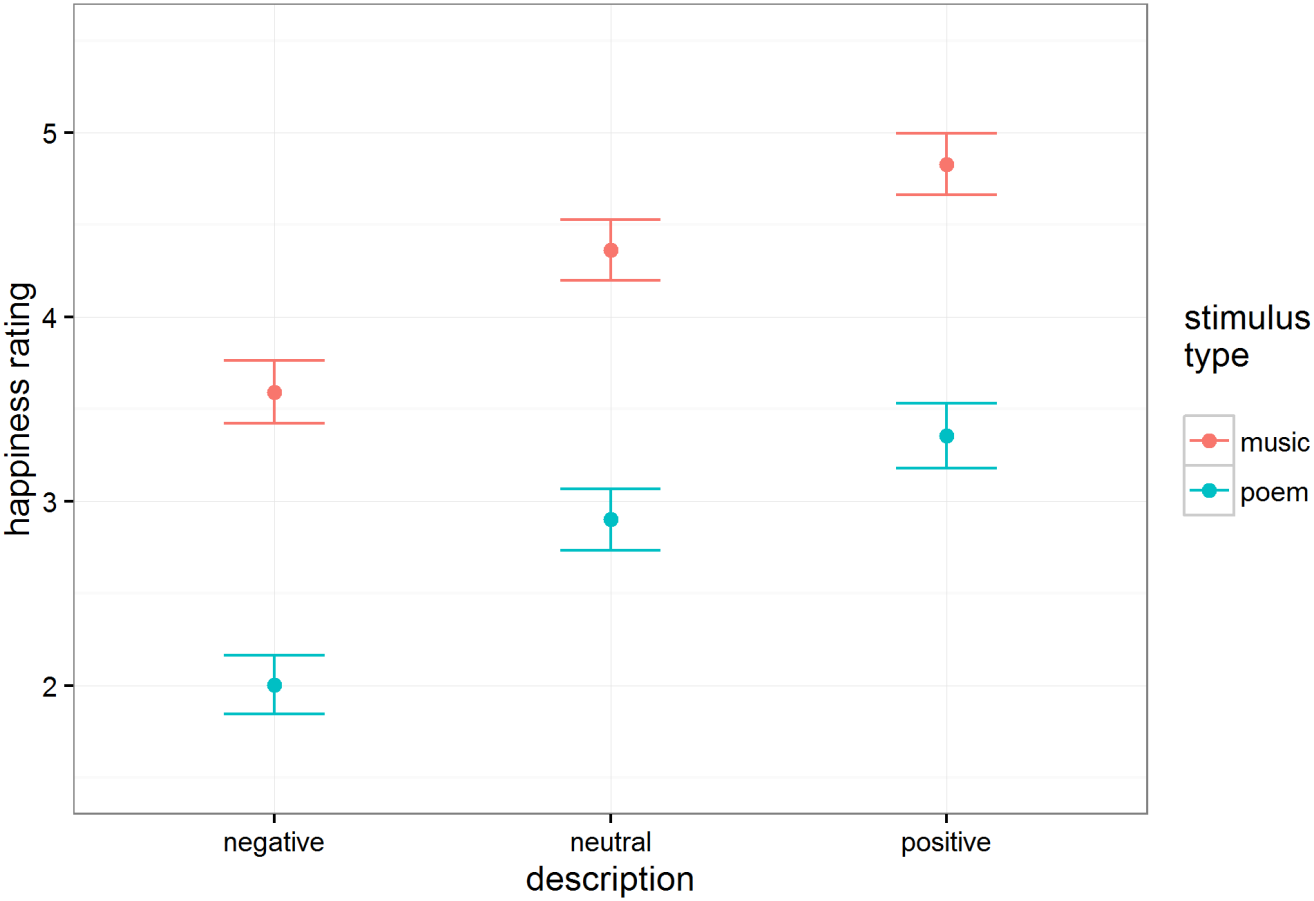
Estimated mean happiness ratings (n = 118 participants) as a function of intention description and stimulus type. Error bars are standard errors.

Happiness ratings appear in Fig 2. There was a clear, predictable effect of intentions, F(2, 154.0) = 199.46, p < .001, such that positive intentions led to an increase in happiness ratings relative to neutral descriptions, and negative intentions led to a decrease in happiness ratings relative to neutral descriptions. Music (M = 4.26, SE = 0.16) elicited higher happiness ratings than poetry overall (M = 2.75, SE = 0.16), t(43.2) = 6.47, p < .001. The two factors did not interact significantly (F < 1).

#### Sadness

**Fig 3:**
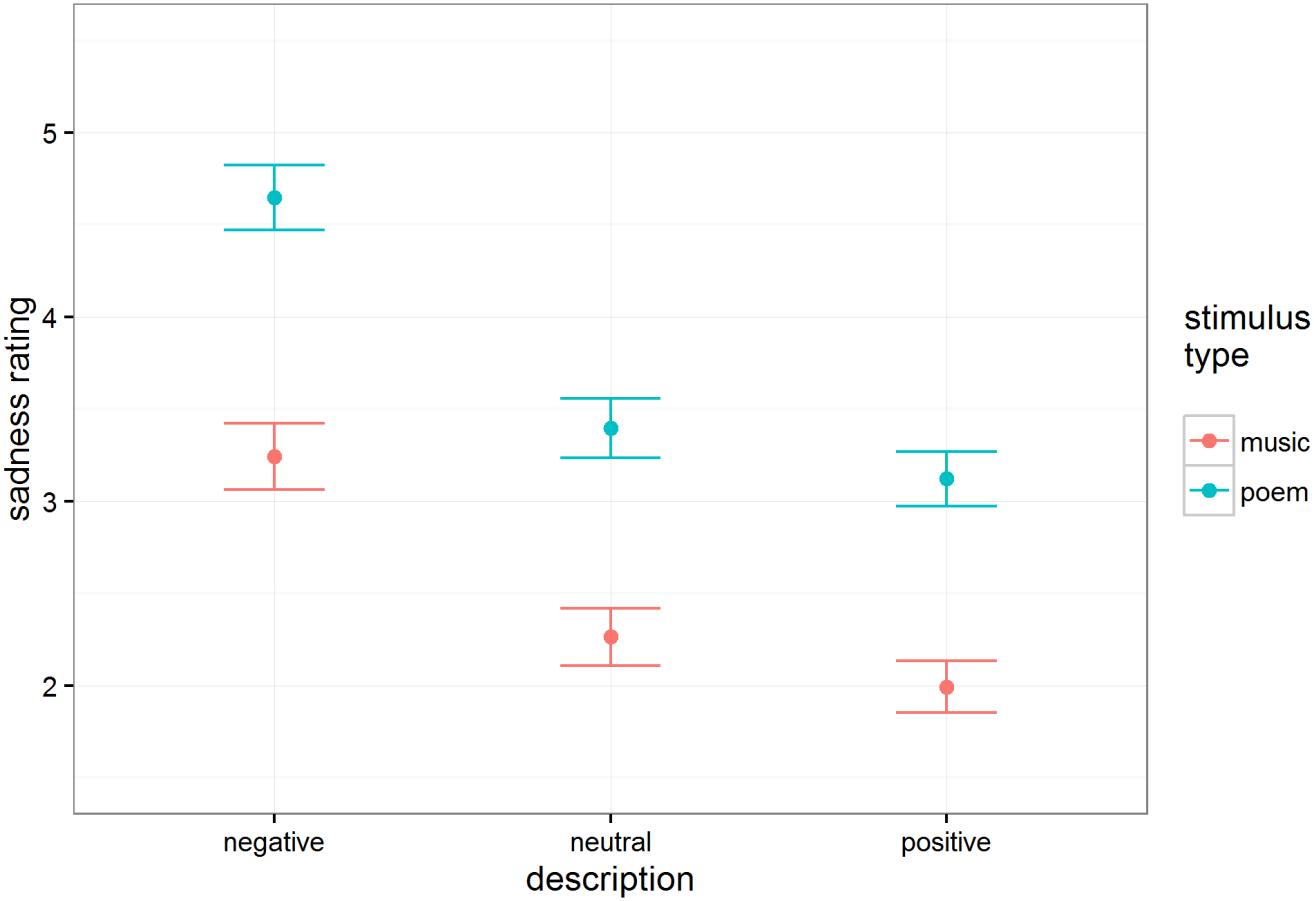
Estimated mean sadness ratings (n = 118 participants) as a function of intention description and stimulus type. Error bars are standard errors.

Sadness ratings mirror those of happiness, as depicted in Fig 3. Again, there was a clear, predictable effect of intentions, F(2, 71.4) = 106.19, p < .001, such that negative intentions led to an increase in sadness ratings relative to neutral descriptions, and positive intentions led to a decrease in sadness ratings relative to neutral descriptions. Poetry (M = 3.72, SE = 0.15) elicited higher sadness ratings than music overall (M = 2.50, SE = 0.15), t(38.5) = 5.31, p < .001. The two factors did not interact significantly (F ≈ 1.5).

#### Movingness

**Fig 4:**
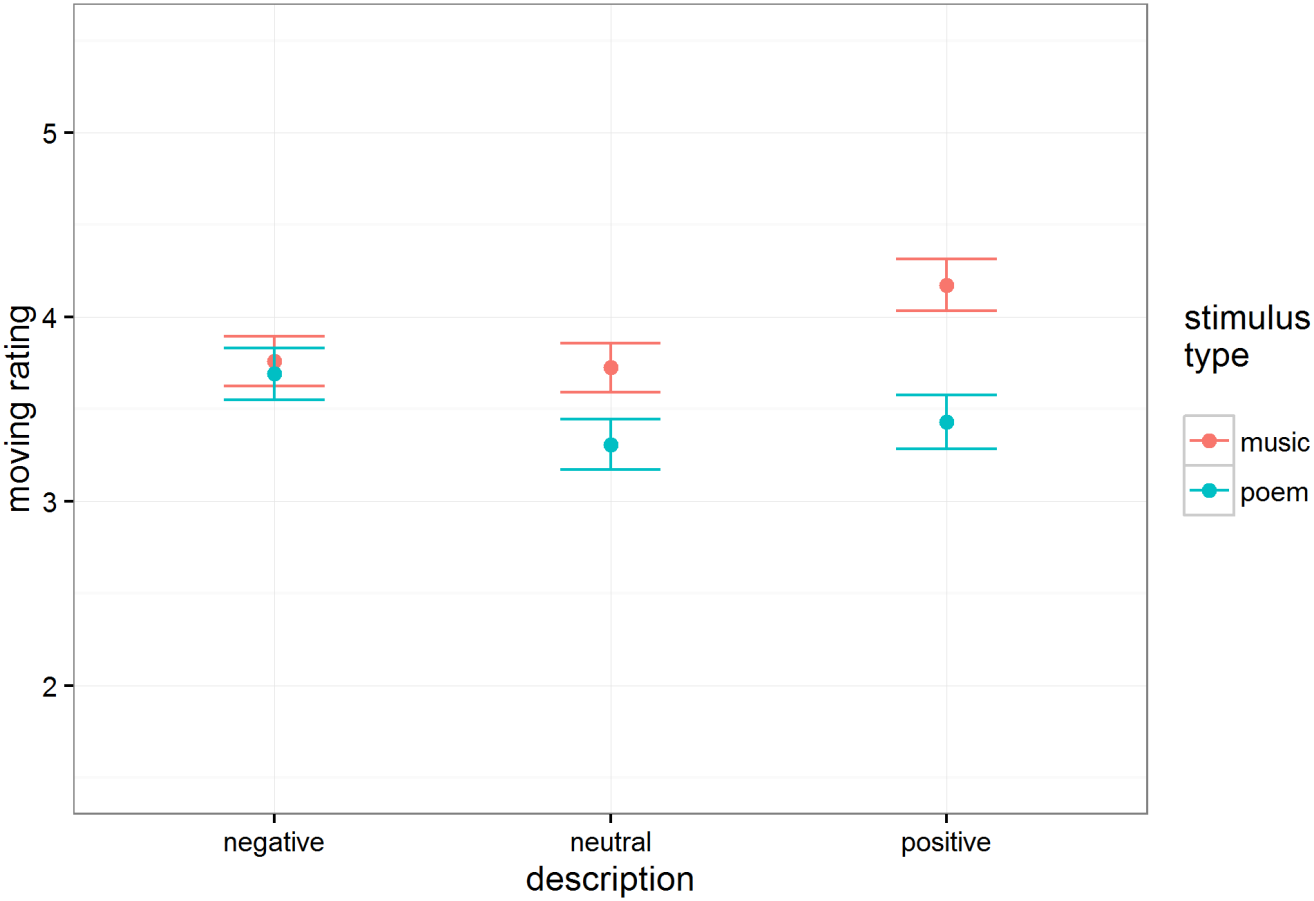
Estimated mean movingness ratings (n = 118 participants) as a function of intention description and stimulus type. Error bars are standard errors.

Movingness ratings showed a distinct difference between the way music and poetry were experienced depending on the intention description's valence (see Fig 4). There was a significant effect of intention description type on movingness, F(2, 57.5) = 16.82, p < .001; both negative (M = 3.73, SE = 0.11) and positive (M = 3.80, SE = 0.11) intentions led to higher movingness ratings than did neutral descriptions (M = 3.52, SE = 0.11). This pattern is qualified by an interaction of intention and excerpt type, F(2, 54.4) = 12.15, p < .001; this interaction reflects that positive intentions increased movingness for music not for poetry, and negative intentions increased movingness for poetry but not for music. Music (M = 3.89, SE = 0.13) was rated as more moving than poetry overall (M = 3.48, SE = 0.13), t(50.2) = 2.48, p = .02.

#### Mediation of enjoyment by match

Following the Selig and Preacher's [61] Monte Carlo method for assessing mediation, we carried out analyses to test if the interaction of intention description with excerpt type on enjoyment was mediated by the perceived match between ambiguous excerpts and the intention description they were paired with. To do this, the analyses reported above for enjoyment were repeated with match as an additional predictor (i.e., covariate) in the regression model. Recall that the interaction of intention description with excerpt type on perceived match in ambiguous excerpts (see "Excerpt selection & description checks") has already been established, a critical step in conventional mediation analyses [62].

For enjoyment, the interaction of intention description with excerpt type is decomposed in Table 1. This table displays the unmediated effects of positive and negative intention descriptions relative to neutral descriptions separately for music and poetry. Relative to neutral intentions descriptions, negative descriptions reduced enjoyment for music but increased enjoyment for poetry. Controlling for match, the intention description by excerpt interactions for enjoyment is no longer significant, F(2, 183.5) = 1.82, p = .16,

**Table 1:** Effects of positive and negative intention descriptions on enjoyment (relative to neutral descriptions).

	unmediated	controlling for match
music		
positive–neutral	0.15 (0.10)	0.004 (0.07)
negative–neutral	-0.23* (0.08)	-0.001 (0.07)
poetry		
positive–neutral	0.11 (0.09)	0.16* (0.07)
negative–neutral	0.27** (0.08)	0.002 (0.07)

Note.

*p < .05.

**p < .001. *SE*s appear in parentheses.

Specifically, ambiguous music was significantly less enjoyable (M = 3.88, SE = 0.13) when paired with negative intention descriptions than when paired with neutral intention descriptions (M = 4.11, SE = 0.14), a difference of -0.232 (SE = 0.077), t(90.7) = -3.03, p = .003; but when controlling for match between description and excerpt, the same comparison was negligible (0.004, SE = 0.068), suggesting complete mediation, supported by a 95% CI for the indirect effect on enjoyment of negative descriptions (relative to neutral descriptions) via match of [-0.308, -0.164]. Similarly, ambiguous poetry when paired with negative intention descriptions was significantly more enjoyable (M = 3.46, SE = 0.141) than when paired with a neutral intention descriptions (M = 3.18, SE = 0.150), a difference of 0.273 (SE = 0.080), t(98.2) = 3.44, p < .001; when controlling for match, the same comparison was negligible (0.002, SE = 0.073), again suggesting complete mediation, supported by a 95% CI for the indirect effect on enjoyment of negative descriptions (relative to neutral descriptions) via match of [0.213, 0.331].

## Discussion

From these data, it is clear that the presentation of information about the artist’s expressive intent influenced people’s emotional experiences of both music and poetry. The presentation of positively valenced information caused people to experience excerpts as happier and less sad. The presentation of negatively valenced information, on the other hand, caused people to experience excerpts as sadder and less happy. These effects were robust and of a similar size for both music and poetry. Given that both domains seem susceptible to the impact of information about expressive intent, an interesting question arises about whether similarities exist between the mechanisms that give rise to aesthetic experiences in both domains. Menninghaus et al. [45], for example, suggests that parallelistic structure, a feature long identified as important to music [63] also shapes aesthetic response for poetry.

The ability of musical and poetic excerpts to so easily take on the emotional tenor suggested by brief statements of authorial or compositional intent confirms prior suggestions [36] that empathy with a perceived human producer is an important part of the emotional experience of art across various media. It also suggests that people are able to integrate verbal information into the aesthetic experience with comparable success regardless of whether the materials of the medium are or are not verbal themselves—that is, people could integrate intent descriptions with experiences of a language-based art (poetry) as well as they could with experiences of a non-language-based art (music). The ability of information provided before an aesthetic experience to alter the way it is processed has been demonstrated by Kroger and Margulis [17] for information about quality, Margulis [18] for information about content, and Brattico et al. [64] for task—ERP evidence demonstrates that people apprehend tonal structure differently depending on whether they are tasked with making a cognitive judgment (whether a particular chord is correct or incorrect) or an affective one (whether they like the chord or not). Future work that further traced the timeline and mechanisms by which top-down information of this sort impacts the perception and evaluation of aesthetic entities would be especially welcome.

Despite that people assimilated expressive information similarly for both media—positive and negative descriptions had similar effects on happy and sad ratings for music and poetry—the baseline happy and sad ratings for music and poetry were clearly different. Regardless of intent information type, musical excerpts were perceived as more happy and less sad, and poetry excerpts were perceived as more sad and less happy. Fascinatingly, this increase in perceptions of sadness when words are involved might extend even to music with lyrics. Brattico et al. [65] showed that happy music without lyrics was perceived as more positive than happy music with lyrics. Although stimulus properties could be an explanatory factor for the results, one possible implication of this difference is that people read more happiness into ambiguous music and more sadness into ambiguous poetry—future work could investigate whether this effect holds for more stimuli and more populations. If so, a possible explanation—given past work showing a general preference for happy over sad music [51], [52]—might be that people listen more frequently to happy music, leading them to use base rate information to assimilate ambiguous excerpts into their ordinary experience by assuming they are happy. Given other findings from this experiment suggesting that people prefer poetry when it has been disambiguated as sad, it might be that people more frequently read sad poetry, leading them to assimilate ambiguous excerpts into their ordinary experience by assuming they are sad. Neuroimaging work shows selective activation in parts of the auditory cortex when listening to happy rather than sad music, suggesting that people may pay more attention to the sensory signal when listening to music they identify as happy [66].

Although positively and negatively valenced intent information influenced emotional experience (happiness and sadness ratings) similarly for music and poetry, it influenced the aesthetic dimensions of the experience (enjoyment and being moved) differently. Positive intent information elevated enjoyment and movingness ratings for music, but negative intent information elevated enjoyment and movingness ratings for poetry. In other words, people’s most powerful aesthetic experiences were reserved for music that had been expressively disambiguated as positive, but for poetry that had been expressively disambiguated as negative.

In general, people reported enjoying the ambiguous musical excerpts more than the ambiguous poetry. They were also generally more moved by the music than by the poems, with the exception of excerpts prefaced by negative intent information—only in this case did people find the poems as moving as music.

Together, these findings suggest that people want their music happy but their poetry sad. This difference may reflect a distinction in the typical social function of these art forms. Music is often listened to in a group setting, and its capacity to facilitate social bonding [67] has been identified as a key characteristic. Music can elicit a sense that boundaries have been dissolved and the listener is physically participating with the sound in some virtual, imagined way [68], [63]. Poetry, on the other hand, is often read in solitude with the goal not of euphorically transcending boundaries and syncing with a group (as may be the case for some music listening), but rather with the goal of achieving insight into human experience [69]. (Note, however that these are broad generalizations, and there are no doubt listeners who approach music in the personal and intimate manner that is more typical of poetry, and vice versa.) Also broadly speaking, people tend to view poetry as challenging and edifying, and may have perceived the excerpts preceded by negative intent information as more serious, and more capable of fulfilling this role. The participants in this study were drawn from students in a General Psychology class at an American state university, and were not selected for having any special interest or expertise in either music or poetry. Given the evidence for how differently experts in these domains process information in their area of expertise [70], and the different goals and criteria those kinds of listeners would bring to the experience, it would be interesting to run the same study using expert poets and musicians as participants.

The positive intent information may have mapped more readily onto music’s most widely presumed function: to elevate mood [71]. Contrastingly, the negative intent information may have positioned the poetry to serve as the kind of deep or thought-provoking artwork people expect from this genre [72], [73]. This interpretation is bolstered by the fact that people thought the positive intent information matched the musical excerpts best, but that the negative intent information matched the poetry excerpts best.

For neither of the categories of artworks were the ambiguous excerpts preferred. On the contrary, disambiguation in the direction most associated with the genre (positive for music, negative for poetry) produced the strongest increases in enjoyment and movingness. Although theoretical approaches have often extolled the value of ambiguity in creating rich, relevant, and multiply-interpretable works of art, this benefit does not seem to extend to expressive ambiguity. Instead, people seem to report more satisfying aesthetic experiences in response to works of art whose primary expressive cast is clear. Since valenced information may make it easier to empathize with the author or composer, this finding supports theories that attribute aesthetic power in part to empathy with a perceived human creator [37], [40], [1].

The opposing roles of positive and negative intent information in music and poetry, however, suggests that the way empathy with a perceived human artist feeds into aesthetic appreciation differs across media. In the case of music, it may allow the listener to relax and experience a sense of shared subjectivity with an implied social group—a process that would likely be more difficult if the expressive tenor were negative, since negative emotions in group settings tend to raise anxiety that could interfere with percepts of successful bonding. In the case of poetry, it may allow the reader to formulate a sense that intimate sensibilities have been conveyed directly from one person to another—from the poet to the reader—without invoking an imagined larger group. Yet, because multiple modes of aesthetic attending are possible, this potential explanation requires further exploration. Together with other recent work, including [24], this study argues for the importance of further work on domain generality and domain specificity in aesthetic attending.

## Supporting information

S1 Appendix Poetry excerpts used as stimuli. (DOCX)

S2 Appendix Music excerpts used as stimuli, from [15]. (DOCX)

S3 Appendix Intent descriptions used for poetry and music. (DOCX)
