# Supplementary material for: Expressive intent, ambiguity, and aesthetic experiences of music and poetry
Source: PLoS One. 2017 Jul 26;12(7):e0179145. doi: 10.1371/journal.pone.0179145 (PMC5528260; doi:10.1371/journal.pone.0179145)
Supplement: S1 Appendix — (DOCX) [file pone.0179145.s001.docx]

| Author | Title | Start Line |
| --- | --- | --- |
| Ambiguous Excerpts | | |
| Adrienne Rich | A Woman Mourned by Daughters | What is it, if not you |
| Andrea Hollander Budy | This | This |
| Dante Gabriel Rossetti | The Woodspurge | The wind flapp’d loose, the wind was still |
| Elizabeth Bishop | The Shampoo | The still explosions on the rocks |
| Ralph Waldo Emerson | Woodnotes I/3 | He heard when in the grove, at intervals |
| Jane Hirshfield | Husband | Some things can surprise you in both directions, |
| John Keats | “Bright star, would I were stedfast as thou art” | Bright star, would I were stedfast as thou art |
| John Masefield | Sea Fever | I must go down to the seas again, for the call of the running tide |
| Mary Ruefle | Inglenook | I live in the museum of |
| National Tarn | Sun’s Clouds | An undulation music of the whale |
| Paul Auster | Autobiography of the Eye | Invisible things, rooted in cold, |
| Michael Drayton | Idea | Shake hands forever, cancel all our vows, |
| Wallace Stevens | The Snow Man | One must have a mind of winter |
| Walt Whitman | A child said, What is the grass | Tenderly will I use you curling grass |
| Emily Dickson | I Should Not Dar | I should not dare to leave my friend, |
| Ernest Dowson | A Last Word | Despair and death; deep darkness o’er the land |
| Edgar Allan Poe | Spirits of the Dead | The spirits of the dead who stood |
| Pablo Neruda | Ode to Bird Watching | Now let’s look for birds! |
| Negative Excerpts | | |
| Elinor Wylie | Cold Blooded Creatures | Man, the egregious egoist |
| Langston Hughes | Kids Who Die | Of course, the wise and the learned |
| Chidiock Tichborne | [My prime of youth is but a frost of cares] | My prime of youth is but a frost of cares, |
| Kim Hooten | Revenge | Tears the mind can’t tell are true |
| Positive Excerpts | | |
| Ralph Waldo Emerson | To laugh often and much | To laugh often and much |
| Ella Wheeler Wilcox | Joy | My heart is like a little bird |
| William Wordsworth | She Was a Phantom of Delight | She was a Phantom of delight |
| Zora Bernice May Cross | Girl-Gladness | So, dear come along |
