# Supplementary material for: Expressive intent, ambiguity, and aesthetic experiences of music and poetry
Source: PLoS One. 2017 Jul 26;12(7):e0179145. doi: 10.1371/journal.pone.0179145 (PMC5528260; doi:10.1371/journal.pone.0179145)
Supplement: S2 Appendix — (DOCX) [file pone.0179145.s002.docx]

| Title | Artist/Composer | Album | Start Time |
| --- | --- | --- | --- |
| Ambiguous Music Excerpts | | | |
| El Monte | Zafra | Caliente: Musica Cubana | 0:04 |
| Sega | Ali Farka Toure with Ry Cooder | Talking Timbuktu | 0:29 |
| Moonlight Serenade | Glenn Miller | His Greatest Hits & Finest Performances | 0:00 |
| Cello Concerto in G Major, Second Movement | Vivaldi | Famous Concertos for Two Trumpets, Oboe, Violins, Cello, Mandolin | 0:37 |
| Concerto for Flute and Harp in C | Mozart | Mozart for Meditation | 0:38 |
| Cornflake Girl | Tori Amos | Under the Pink | 2:25 |
| Organ Donor | DJ Shadow | Pysence Fiction | 0:11 |
| Exit Music (For a Film) | Christopher O’Riley | True Love Waits | 0:52 |
| Billy Peddle | Great Big Sea | Up | 0:13 |
| Concerto for Piano and Trumpet No. 1 in C Minor, First Movement | Shostakovich | Concerto For Piano and Trumpet No .1,  Piano Concerto No. 2, Trio No. 2 | 2:43 |
| Symphony No. 6, Second Movement | Beethoven | On A Classical Note: Beethoven Symphony  No. 5, Symphony No. 6—‘‘Pastoral’’ | 1:12 |
| (Past Due) | The Weakerthans | Reconstruction Site | 1:15 |
| La Noyee | Yann Tiersen | Amelie Soundtrack | 0:08 |
| There There | Radiohead | Hail to the Thief | 0:12 |
| Quien hubera dicho | Silvanan Deluigi | Tanguera: Woman in Tango | 1:54 |
| Untitled 4 | Sigur Ros | () | 0:48 |
| Protection | Massive Attack | Protection | 5:31 |
| Plainsong | The Cure | Disintegration | 1:29 |
| Positive Music Excerpts | | | |
| Symphony No. 6, First Movement | Beethoven | On A Classical Note: Beethoven Symphony  No. 5, Symphony No. 6—‘‘Pastoral’’ | 3:08 |
| Loyin Loyin | Babatunde Olatunji | Best of Both Worlds: The Rykodisk World  Music Sampler | 0:24 |
| Santiago | Zafra | Caliente: Musica Cubana | 0:04 |
| Concerto (Sinfonia) in D Major, First Movement | Vivaldi | Famous Concertos for Two Trumpets, Oboe, Violins, Cello, Mandolin | 0:01 |
| Negative Music Excerpts | | | |
| Piano Concerto No. 23 in A, Adagio | Mozart | Mozart for Meditation | 0:53 |
| Icicle | Tori Amos | Under the Pink | 0:18 |
| Rabbit in your Headlights (3DMix Reverse Light) | UNKLE | Rabbit in Your Headlights | 6:24 |
| Fake Plastic Trees | Christopher O’Riley | True Loves Waits | 0:04 |
