# Supplementary material for: Expressive intent, ambiguity, and aesthetic experiences of music and poetry
Source: PLoS One. 2017 Jul 26;12(7):e0179145. doi: 10.1371/journal.pone.0179145 (PMC5528260; doi:10.1371/journal.pone.0179145)
Supplement: S3 Appendix — (DOCX) [file pone.0179145.s003.docx]

| Positive descriptions |
| --- |
| The author/composer wrote this poem/piece to express passion for a lover |
| The author/composer wrote this poem/piece to capture the beauty of an immaculate sunrise |
| The author/composer wrote this poem/piece to rejoice in spiritual devotion |
| The author/composer wrote this poem/piece in response to an amazing experience with a work of art |
| The author/composer wrote this poem/piece before embarking on an exciting journey |
| The author/composer wrote this poem/piece to applaud a daughter’s inspiring success |
| The author/composer wrote this poem/piece in a time of great personal happiness |
| The author/composer wrote this poem/piece to rejoice in a dear friend’s good fortune |
| The author/composer wrote this poem/piece to celebrate a sister’s wedding |
| The author/composer wrote this poem/piece to communicate hope for the New Year |
| The author/composer wrote this poem/piece after reuniting with a favorite childhood friend |
| The author/composer wrote this poem/piece to express joy at the birth of a long-awaited-for child |
| Negative descriptions |
| The author/composer wrote this poem/piece to convey sadness about a friend’s addiction |
| The author/composer wrote this poem/piece after spending many years in isolation from friends and family |
| The author/composer wrote this poem/piece after returning from active duty in a war |
| The author/composer wrote this poem/piece while hospitalized after an accident |
| The author/composer wrote this poem/piece after witnessing widespread poverty |
| The author/composer wrote this poem/piece to grieve the loss of an unborn child |
| The author/composer wrote this poem/piece to mourn the death of a friend |
| The author/composer wrote this poem/piece to capture the horrifying aftermath of a natural disaster |
| The author/composer wrote this poem/piece during a difficult period of political unrest |
| The author/composer wrote this poem/piece during a long period of dreary and depressing weather |
| The author/composer wrote this poem/piece after a daughter lost her job |
| The author/composer wrote this poem/piece in response to a parent’s illness |
| Neutral descriptions |
| The author/composer wrote this poem/piece to mimic the voice of another author/composer |
| The author/composer wrote this poem/piece as a contribution to a new collection |
| The author/composer wrote this poem/piece to fulfill a commission from a wealthy patron |
| The author/composer wrote this poem/piece to experiment with different writing techniques |
| The author/composer wrote this poem/piece to model particular styles for a group of students |
| The author/composer wrote this poem/piece in two days |
| The author/composer wrote this poem/piece for simultaneous release in several countries |
| The author/composer wrote this poem/piece as preparation for writing a longer piece |
| The author/composer wrote this poem/piece for a public event |
| The author/composer wrote this poem/piece as an exercise between writing two lengthy works |
| The author/composer wrote this poem/piece to express feelings toward a community |
| The author/composer wrote this poem/piece in order to learn about a different era |
